# Spontaneous mutation rate estimates for the principal malaria vectors *Anopheles coluzzii* and *Anopheles stephensi*

**DOI:** 10.1038/s41598-021-03943-z

**Published:** 2022-01-07

**Authors:** Iliyas Rashid, Melina Campos, Travis Collier, Marc Crepeau, Allison Weakley, Hans Gripkey, Yoosook Lee, Hanno Schmidt, Gregory C. Lanzaro

**Affiliations:** 1 Vector Genetics Laboratory, Department of Pathology, Microbiology and Immunology, UC Davis, 1089 Veterinary Medicine Dr, 4225 VM3B, Davis, CA 95616 USA; 2 Section of Cell and Developmental Biology, University of California, San Diego, La Jolla, CA USA; 3 Tata Institute for Genetics and Society, Center at inStem, Bangalore, Karnataka 560065 India; 4 Department of ChEM-H Operations, Stanford University, 450 Serra Mall, Stanford, CA 94305 USA; 5 Florida Medical Entomology Laboratory, University of Florida, 200 9th St SE, Vero Beach, FL 32962 USA; 6 Anthropology, Institute of Organismic and Molecular Evolution (iomE), Johannes Gutenberg University of Mainz, Saarstraße 21, 55122 Mainz, Germany

**Keywords:** Evolutionary genetics, Evolutionary biology, Mutation, Sequencing, Entomology

## Abstract

Using high-depth whole genome sequencing of F0 mating pairs and multiple individual F1 offspring, we estimated the nuclear mutation rate per generation in the malaria vectors *Anopheles coluzzii* and *Anopheles stephensi* by detecting de novo genetic mutations. A purpose-built computer program was employed to filter actual mutations from a deep background of superficially similar artifacts resulting from read misalignment. Performance of filtering parameters was determined using software-simulated mutations, and the resulting estimate of false negative rate was used to correct final mutation rate estimates. Spontaneous mutation rates by base substitution were estimated at 1.00 × 10^−9^ (95% confidence interval, 2.06 × 10^−10^—2.91 × 10^−9^) and 1.36 × 10^−9^ (95% confidence interval, 4.42 × 10^−10^—3.18 × 10^−9^) per site per generation in *A. coluzzii* and *A. stephensi* respectively. Although similar studies have been performed on other insect species including dipterans, this is the first study to empirically measure mutation rates in the important genus *Anopheles*, and thus provides an estimate of µ that will be of utility for comparative evolutionary genomics, as well as for population genetic analysis of malaria vector mosquito species.

## Introduction

The process of evolution depends on the occurrence of new mutations which provide genetic variation and influence phenotypic traits^1^. The rate of de novo mutations is a key determinant of the rate of evolution of an organism under a molecular clock model^2^. DNA repair mechanisms normally ensure that genetic material is copied with fidelity during meiosis and transferred from one generation to the next^3^. However, at some small frequency, the transmission of damaged and improperly repaired DNA, or of DNA with replication errors, to the next generation causes a germline mutation in the offspring, which may then be subject to natural selection^4^. The precise estimation by empirical methods of the de novo germline mutation rate in multicellular organisms with large genome sizes has remained a great challenge even with the advent of next-generation DNA sequencing technologies because of inherent limitations, biases, and errors^5^.

Previous studies have been conducted to estimate the mutation rate per generation in organisms ranging from prokaryotes to eukaryotes including invertebrates and vertebrates^6^. The rate of the mutation per site per generation in vertebrates was estimated as 4.6 × 10^–9^ in a bird species (the flycatcher *Ficedula albicollis*)^7^, 4.5 × 10^–9^ in wolves (*Canis lupus)*^8^, and 5.4 × 10^−9^ in mouse (*Mus musculus)*^9^. The spontaneous mutation per base pair per generation for African green monkeys (*Chlorocebus sabaeus*)^10^ was estimated at a rate of 0.94 × 10^–8^ which is slightly lower than the rate of 1.2 × 10^–8^ per base pair per generation that was reported in chimpanzees (*Pan troglodytes*)^11^. The mutation rate per base per generation estimated in humans was 1.2 × 10^−8^ in two separate studies^12,13^ and was associated with a higher mutation rate in exons compared to introns^14^. Recent studies reveal that many factors may affect mutations rates, including genomic heterogeneity, population differences, and both *cis-* and *trans-*acting factors that influence mutagenic processes^15–17^.

In invertebrate species, Keightley et al. estimated a similar mutation rate per generation between two insect species in consecutive studies: *Drosophila melanogaster*^18^ at 2.8 × 10^−9^ and *Heliconius melpomene*^19^ at 2.9 × 10^−9^. Likewise, estimated mutation rates in two bee species were very close. Honeybee (*Apis mellifera*)^20^ was estimated at 3.4 × 10^−9^ and bumblebee (*Bombus terrestris*)^21^ at 3.6 × 10^−9^ per haploid genome, which is approximately 20–24% higher than *H. melpomene* and *D. melanogaster*. The rates for the two bee species were nearly identical despite a ~ 4-fold difference in recombination rate. Oppold and Pfenninger^22^ presented a mutation rate per generation for a non-biting midge, *Chrironomus riparius*, at the lower range of other insect rates (2.1 × 10^−9^). These reports in insects laid the groundwork for our study of mosquitoes reported here.

Female mosquitoes of the genus *Anopheles* are well known as malaria vectors, but not all species within the genus are efficient transmitters of human malaria. *Anopheles coluzzii* and *Anopheles stephensi* are two of the most intensively studied mosquito species. This focus is because they are the main human malaria vectors, the former in Africa and the latter in Asia, mainly in the Indian subcontinent region and Middle East^23,24^. The infected female *Anopheles* mosquito is responsible for transmitting the malaria parasites (*Plasmodium* species) to people through their bites^25^. Characteristically, *Anopheles* mosquitoes mate in flight within a swarm where groups of male mosquitoes gather and attract virgin females at dusk. Typically, a single male mosquito copulates with each female^26,27^. The karyotype of anopheline mosquitos is comprised of one pair of sex chromosomes (XX for female and XY for male) and two pairs of submetacentric autosomes. The longer arm is designated the right arm and the autosomal compliment is typically designated R and L thus yielding 2R, 2L and 3R, 3L^28^.

This study obtained mutation rate estimations for *A. coluzzii*, an Afrotropical malaria mosquito belonging to the *Anopheles gambiae* species complex^29^, and *A. stephensi,* an urban malaria vector widespread in Asia^24^ and recently emerging as a major malaria vector, with life-threatening potential, in east Africa^30,31^. The *A. gambiae* complex is comprised of at least nine closely related, homomorphic sibling species, varying in their geographical distributions^32,33^. Some act as dominant vectors of malaria, some as secondary vectors^34^, and others are non-vector species^23^. Like other sibling species of this group, *A. coluzzii*, plays a crucial role in human malaria transmission in west and central Africa^35,36^ and its genome has been intensively studied^37^.

Several approaches have been applied for the estimation of mutation rates in eukaryotes, including indirect methods that utilize established principles of population genetic theory to infer and extrapolate from polymorphisms detected in sequenced genomic subregions^38–40^. The advent of next-generation sequencing (NGS) established a new modality for the identification of de novo mutations by applying whole-genome sequencing to a pedigree, followed by a site-by-site genomic comparison of parents and offspring^8,20,21^. The observation of de novo mutations from high depth genome sequencing of parents and offspring is a conceptually simple and effective approach, although it is complicated by errors intrinsic to the nature of the NGS data and its analysis^41,42^. Here, we adopted an insect-favorable method for direct detection of mutations and estimation of the base substitution rate per generation using parent-offspring, multi-fold genome sequencing as initially proposed by Keightley et al.^18^. This is an effective approach of direct estimation by categorizing a large set of offspring into two classes, focal and bait, to minimize false positive mutations from the variant call set^19^.

In the current study, we sequenced the genomes of parents and numerous offspring of the malaria mosquitos *A. coluzzii* and *A. stephensi*. Variant calls generated from the sequence dataset for each species were then examined for evidence of point mutations by applying a series of filtering criteria to exclude false positives. Estimation of de novo mutation rate per generation in two *Anopheles* species can illustrate variation in mutation rates across species in this important group of mosquitos and can offer an open path for the interpretation of the molecular clock and demographic history.

## Results

Successful mating and offspring generation was obtained with a female:male ratio of 1:4 for *Anopheles coluzzii*, and 1:2 for *A. stephensi.* Fatherhood was determined using whole genome sequencing information obtained for each potential male parent. Parental and focal offspring genomes were sequenced at a high coverage (≥ 20), whereas “bait offspring” were sequenced at lower coverage (≤ 20) (Supplementary Tables 1 and 2). One *A. coluzzii* offspring sample (Ac-F1-F13) was removed from analysis due to low yield and poor mappability (< 2%). We obtained 10 focal and 19 bait offspring for *A. coluzzii* and 13 focal and 17 bait offspring for *A. stephensi* (Table 1). Mean fold genome coverage for focal offspring ranged from 22.1 to 33.1 for *A. coluzzii* and from 30.2 to 46.1 for *A. stephensi*. Mean fold genome coverage ranges for the bait offspring were from 8.9 to 18.0 for *A. coluzzii* and from 15.1 to 20.1 for *A. stephensi*. Parents of both *Anopheles* species achieved mean genome coverage of ~ 27 times or higher, except the female parent of *A. coluzzii* which had a comparatively lower coverage mean of 20.6 (Table 1; Supplementary Tables 1 and 2).

**Table 1:** Whole genome sequencing coverage results for each sample categorization.

Species	Sample type	N	Coverage			
			25th	Median	75th	Mean
*Anopheles coluzzii*	Female parent	1	16	21	25	20.4
	Putative male parent	4	24	31	40	32.4
	Focal offspring	10	22.1	26	32	25.7
	Bait offspring	19	8.9	13	17	13.1
*Anopheles stephensi*	Female parent	1	30	36	41	35.7
	Putative male parent	2	22	28	34	28.2
	Focal offspring	13	27	32	38	32.2
	Bait offspring	17	11	14	17	14.4

Number of samples (N) and whole genome coverage results for parents and offspring types (focal and bait) for *A. coluzzii* and *A. stephensi.*

*25th* 25th percentile, *75th* 75th percentile.

### Male parent identification

**Figure 1:**
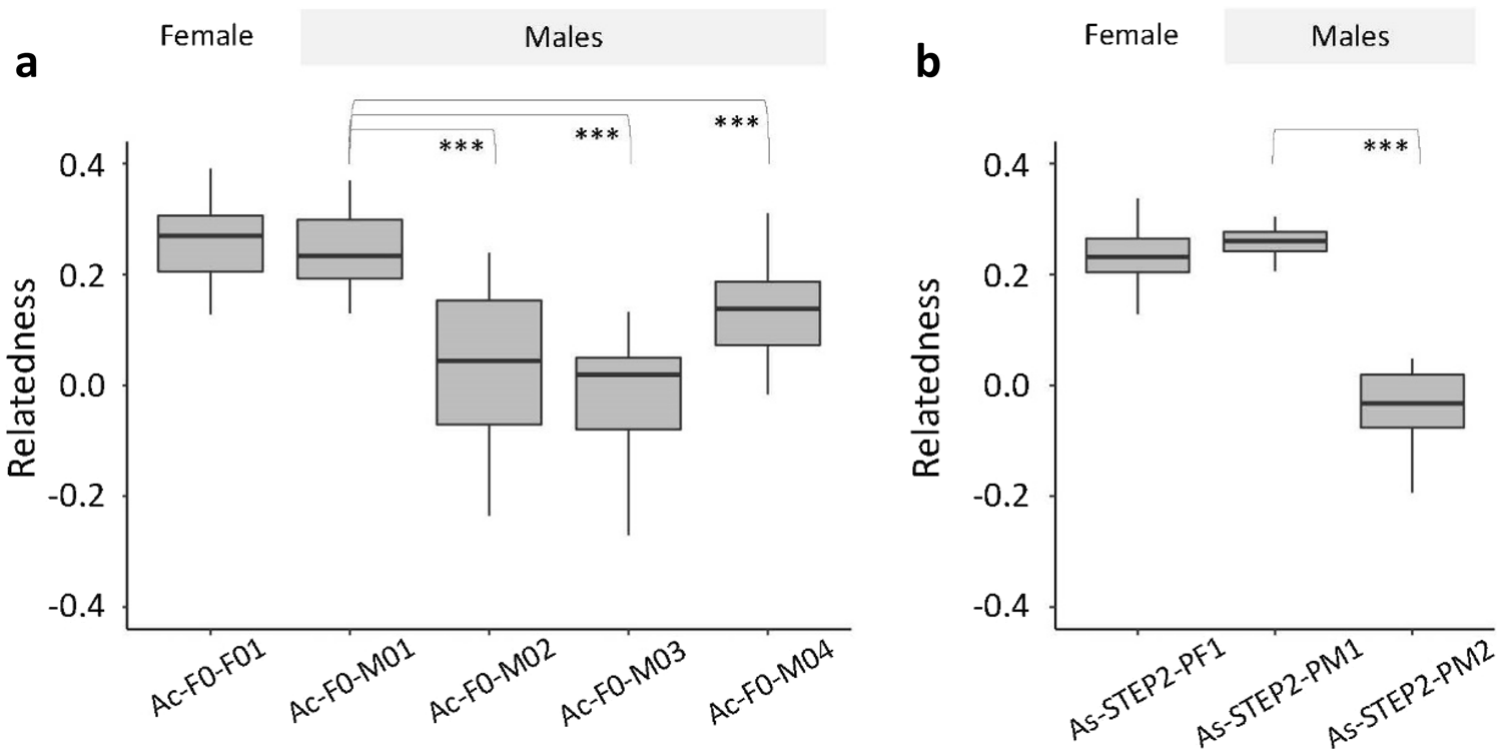
Male parent identification of *A. coluzzii* and *A. stephensi* offspring. Boxplots of relatedness values between each potential male parent and all offspring. Both species had a unique female parent and 4 potential male parents for *A. coluzzii* and 2 for *A. stephensi*. ***p-value < 0.0001; Wilcoxon rank-sum test.

After joint variant calling for each species, true fathers were identified by relatedness^43^ and Mendelian inheritance-based analysis, both using only autosomal, biallelic SNPs from all offspring. For *A. coluzzii*, male Ac-F0-M01 presented a significantly higher relatedness with offspring specimens than any other male (*p*-value < 0.0001; Fig. 1a). The same male sample showed the highest proportion of observed heterozygous genotypes among all offspring based on expected heterozygosity, i.e., when both parents are homozygous for different alleles (Table 2). For *A. stephensi*, male As19-STE2-PM1 was closely related to the offspring by both methods: relatedness test (*p*-value < 0.0001; Fig. 1b) and Mendelian analysis (Table 2).

**Table 2:** Mendelian inheritance for male parent identification.

Species	Parent pair (female, male)	Homozygous for different allele	Number of sites	Heterozygous genotypes in all offspring (percentage)
*Anopheles coluzzii*	Ac-F0-F01 Ac-F0-M01	Ref × Alt Alt × Ref	9038 8227	6962 (77.03%) 6584 (80.03%)
	Ac-F0-F01 Ac-F0-M02	Ref × Alt Alt × Ref	40,095 39,555	8 (0.02%) 2 (0.01%)
	Ac-F0-F01 Ac-F0-M03	Ref × Alt Alt × Ref	33,316 28,923	3 (0.01%) 15 (0.05%)
	Ac-F0-F01 Ac-F0-M04	Ref × Alt Alt × Ref	18,591 14,986	5 (0.03%) 3 (0.02%)
*Anopheles stephensi*	As19-STE2-PF1 As19-STE2-PM1	Ref × Alt Alt × Ref	19,740 29,616	16,523 (83.70%) 25,100 (84.75%)
	As19-STE2-PF1 As19-STE2-PM2	Ref × Alt Alt × Ref	30,949 43,070	44 (0.14%) 42 (0.10%)

The Mendelian inheritance-based approach for paternity analysis reveals the number of sites at which all offspring are heterozygous for different pairs of parents where parents are homozygous for different alleles.

### Identification of candidate de novo mutations

The whole-genome variant calling algorithm returned a variant call format file (VCF) for each species. 6,150,332 variants were called for *A. coluzzii*, and 2,556,842 for *A. stephensi*. After filtering for biallelic SNPs, a total of 5,141,452 variant sites were found in *A. coluzzii* and 2,189,630 in *A. stephensi* (Table 3). These were the set of biallelic variant sites used for identification of candidate de novo mutations using a program in Perl that filters each criteria step. The first criterion applied was calling both parents as homozygous for the reference allele with a minimum read depth of 10, which yielded a total of 298,527 and 45,338 variant sites in *A. coluzzii* and *A. stephensi*, respectively (Table 3). Next, variant sites with alternative alleles that were either in the bait offspring or not called in every focal offspring were filtered out, resulting in 256,261 sites for *A. coluzzii*, and 33,203 for *A. stephensi*. From those results, homozygous sites were filtered out leaving 170 heterozygous sites in one or two focal offspring in *A. coluzzii*, and 46 heterozygous sites in *A. stephensi*. Finally, in *A. coluzzii,* 8 autosomal sites were identified as candidate mutations based on their proportion of reference and alternative allele reads, with an average read depth of 33.8 for the parents and 28.3 for all focal offspring (Supplementary Table 3). In *A. stephensi*, 4 candidate mutations were detected on autosomes and two on the X chromosome with an overall average read depth of 30.1 for the parents and 24.7 for all focal offspring (Supplementary Table 4).

**Table 3:** Candidate mutation detection summary.

Filtering site steps	*Anopheles coluzzii*	*Anopheles stephensi*
Total variants	6,150,332	2,556,842
Biallelic SNPs	5,141,452 (83.6%)	2,189,630 (85.6%)
Reference homozygous sites DP ≥ 10	298,527 (4.9%)	45,338 (1.8%)
Sites without alternate allele in bait offspring	277,677 (4.5%)	35,959 (1.4%)
Sites called in every focal offspring	256,261 (4.2%)	33,203 (1.3%)
One or two heterozygous allele in focal	170	49
27% < AAF < 73% on a heterozygous site	8	6

The number of sites filtered out at each step of the candidate mutation detection protocol.

*DP* depth, *AAF *alternate allele frequency.

### Manual curation and validation by Sanger sequencing

After examination on IGV, 9 out of 14 detected candidate mutations were forwarded for Sanger sequencing confirmation: 4 for *A. coluzzii* and 5 for *A. stephensi* (Table 4). Acceptable candidate mutations were found in the heterozygous condition in a single focal offspring and were entirely absent in reads from the parents. Those candidates that were rejected displayed clear evidence of being artifacts of read mis-mapping. Mis-mapping was typically in the form of nearby variants on the same reads that segregated identically with the candidate SNP (Supplementary Figs. 1 and 2). Sanger sequencing for the parent–offspring trio confirmed 3 candidates for *A. coluzzii* and all 5 in *A. stephensi*. Confirmed mutations were all found in non-coding regions. Half of them were transitions and the other half transversions.

**Table 4:** Candidate de novo mutations detected in *A. coluzzii* and *A. stephensi* by whole genome sequencing and validated by Sanger re-sequencing.

Chromosome, position	Ref	Alt	Focal offspring	Allele depth		Mean depth		Confirmed	Location
				Ref	Alt	Parents	Focal		
***Anopheles coluzzii***									
2L, 21501855	A	G	Ac-F1-M01	16	6	35	28.1	No	–
3L, 10978191	G	A	Ac-F1-M04	8	9	31.5	26.9	Yes	Intron
3L, 30182152	A	C	Ac-F1-M05	15	8	31.5	28.2	Yes	Intergenic
3L, 32059779	C	T	Ac-F1-F09	11	7	26.5	17.8	Yes	Intergenic
***Anopheles stephensi***									
X, 1692026	C	T	As19-STE2-F23	18	8	22	18.6	Yes	Intergenic
X, 14920753	C	T	As19-STE2-M15	0	12	27	17.8	Yes	Intron
2, 29186821	T	A	As19-STE2-M09	15	19	39.5	30.8	Yes	Intergenic
3, 6181251	G	C	As19-STE2-M02	13	14	26.5	26.3	Yes	Intron
3, 56020205	G	T	As19-STE2-F14	10	9	35.5	27.2	Yes	Intergenic

Genomic position, reference (Ref.) and alternate (Alt.) alleles in focal offspring and allele depth. Read depth mean for parents and focal offspring. All candidates were re-sequenced by Sanger sequencing for validation.

### Synthetic mutations analysis

We generated synthetic mutations to estimate the false negative rate (FNR) of our mutation detection protocol for each species (see “Methods”). In total, 1000 synthetic mutations were inserted in each of the focal offspring (*A. coluzzii—*10 individuals; *A. stephensi*—13 individuals) across randomly selected autosomal sites where both parents achieved a read depth of 10 or higher. For *A. coluzzii*, of the 10,000 synthetic mutations introduced, 9914 had a read depth of 10 or higher for focal offspring, and therefore these were considered for FNR calculation. Ultimately, our de novo mutation detection program detected 7,658 synthetic mutations that passed all filters. This implies a FNR of 22.8% for the *A. coluzzii* analysis (2,256 synthetic mutations were not detected out of 9,914 inserted in callable sites; Supplementary Table 6). In *A. stephensi*, 12,724 out of 13,000 synthetic mutations were callable, of which 9563 were detected. Therefore, this species presented a FNR of 24.8% (Supplementary Table 6).

### Mutation rate calculation

Our direct sequencing of the offspring of a single mating confined the measurement to a single generation and the calculation was resolved to a simple ratio of mutations per diploid genome corrected for false negatives. We confirmed three de novo mutations in 3.89 × 10^9^ callable sites for *A. coluzzii* and five in 4.88 × 10^9^ sites for *A. stephensi*. Estimated mutation rates for each species were 1.00 × 10^−9^ per site per generation (95% confidential interval, 2.06 × 10^−10^–2.91 × 10^−9^) for *A. coluzzii* and 1.36 × 10^−9^ per site per generation (95% confidential interval, 4.42 × 10^−10^—3.18 × 10^−9^) for *A. stephensi* (Supplementary Table 6).

## Discussion

All eight mutations detected occurred in non-coding regions with equal numbers of transitions and transversions. Five out of the eight de novo mutations detected occurred in male offspring. Male mutation bias in eukaryotes is well known^14,44,45^. Sanger sequencing validation identified only a single false positive mutation in *A. coluzzii* among all the de novo mutations detected by our bioinformatics pipeline. The minimal false-positive rates supported previous studies which showed that higher sequencing depth in multiple samples reduced the false positive rate. These previous studies reveal that 30× and higher read depth data in multiple samples gives 99% genotype accuracy, but lowering read depth increases false-positive genotypes due to mis-mapping and paralogous reads^18,46,47^.

**Figure 2:**
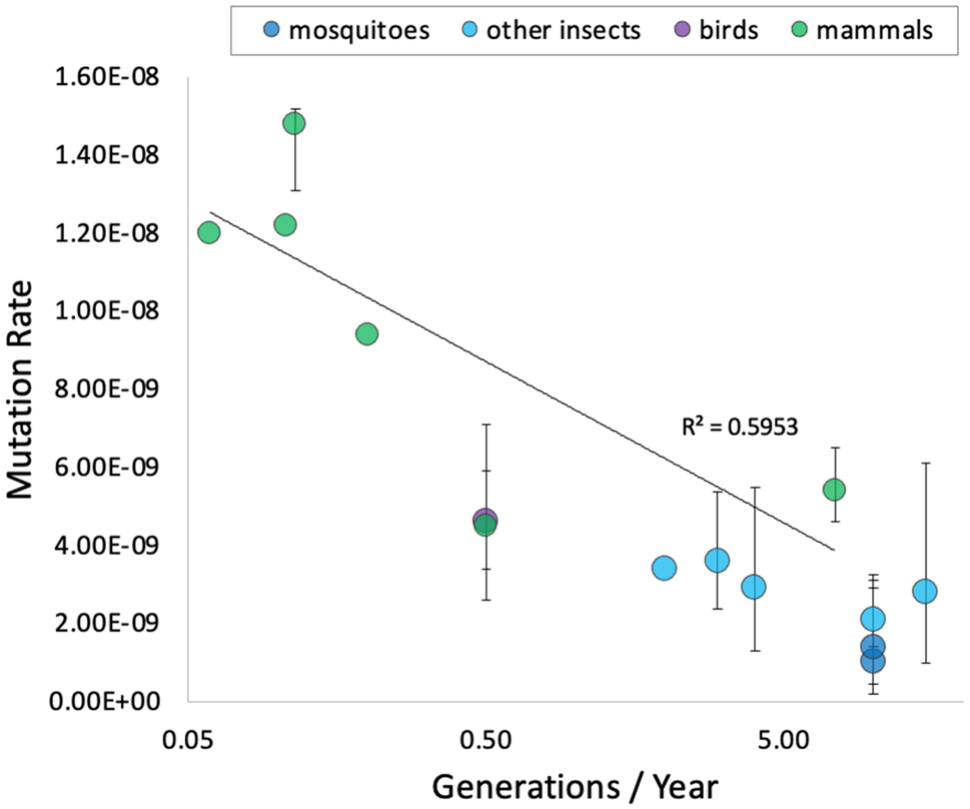
Mutation rate estimation and number of generations per year. Plot of number of generations per year (x-axis) against mutation rate estimations (y-axis) for six species of mammals, green circles (*Canis lupus*, *Mus musculus*, *Chlorocebus sabaeus*, *Gorilla gorilla*, *Pan troglodytes* and *Homo sapiens*), a bird, violet circle (*Ficedula albicollis*), the two species of mosquitoes estimated in this study, blue circles and five non-mosquito insects (*Chironomus riparius*, *Drosophila melanogaster*, *Apis mellifera*, *Bombus terrestris*, *Heliconius melpomene*) cyan circles. Error bars correspond to 95% CI values when provided.

Like reports in bees and insects^18–21^, estimated mutation rates in *A. coluzzii* and *A. stephensi* were similar to each other. Overall, data for mutation rates in bees, flies, and mosquitos confirm that mutation rates are very close among species within the class Insecta, whereas values among mammal species differ by nearly an order of magnitude (Table 5). Interestingly, genome sizes among these eukaryotic species are not similar (Table 5), supporting the suggestion that genome size does not significantly influence mutation events in eukaryotes^7,19,48^. Conversely, there is a negative correlation (R^2^ = 0.5953) between mutation rate and generation time (Fig. 2). Species with long generation times (i.e., few generations per year) tend to have high mutation rates and vice versa, a trend that is apparent across distant taxa from insects to mammals. This might be a mechanism to balance mutation load over evolutionary timescales^49^. The mutation rate of a species is a vital parameter applied in evolutionary and population genetics, but estimating mutation rates is a formidable challenge^48^.

**Table 5:** Available direct estimates of mutation rate in vertebrates and invertebrates.

Species	Mutation rate	Genome size (Mb)	References
**Vertebrates**			
*Homo sapiens*	1.1 × 10^–8^	3232	^41^
*Pan troglodytes*	1.2 × 10^–8^	3309	^11^
*Chlorocebus aethiops*	0.94 × 10^–8^	2797	^10^
*Gorilla gorilla*	1.22 × 10^–8^	3084	^76^
*Canis lupus*	4.5 × 10^–9^	2350	^8^
*Mus musculus*	5.4 × 10^–9^	2671	^9^
*Ficedula albicollis*	4.6 × 10^–9^	1118	^7^
**Invertebrates**			
*Apis mellifera*	3.4 × 10^–9^	247	^20^
*Bombus terrestris*	3.6 × 10^–9^	433	^21^
*Drosophila melanogaster*	2.8 × 10^–9^	148	^18^
*Heliconius melpomene*	2.9 × 10^–9^	269	^19^
*Chironomus riparius*	2.1 × 10^–9^	210	^22^
*Anopheles coluzzii*	1.0 × 10^–9^	280	This study
*Anopheles stephensi*	1.4 × 10^–9^	240	This study

Mutation rate and genome size of different species of vertebrates and invertebrates.

Point mutations occur in both non-coding and coding regions and are typically characterized as neutral^50^, beneficial^51^, or deleterious^52^ based on their effect on fitness. As the names imply, neutral mutations show negligible effects on fitness, beneficial mutations increase the fitness of carriers and their frequencies tend to increase because of positive selection, and deleterious mutations decrease the fitness of carriers and tend to be removed from a population by purifying selection. Mutations in non-coding sequence are not necessarily neutral because they may alter protein binding sites that have direct consequences on gene regulation^53,54^. All mutations in this study were identified in non-coding regions.

The mutation rate per generation in a eukaryotic species is extremely low, meaning that only a few mutations evolve within an individual’s entire genome. Detecting newly risen mutations efficiently is a tedious quest, especially in large genomes. We have found the approach presented herein to be very effective and it is our hope that it will prove useful if applied to other mosquito and insect species.

## Methods

**Figure 3:**
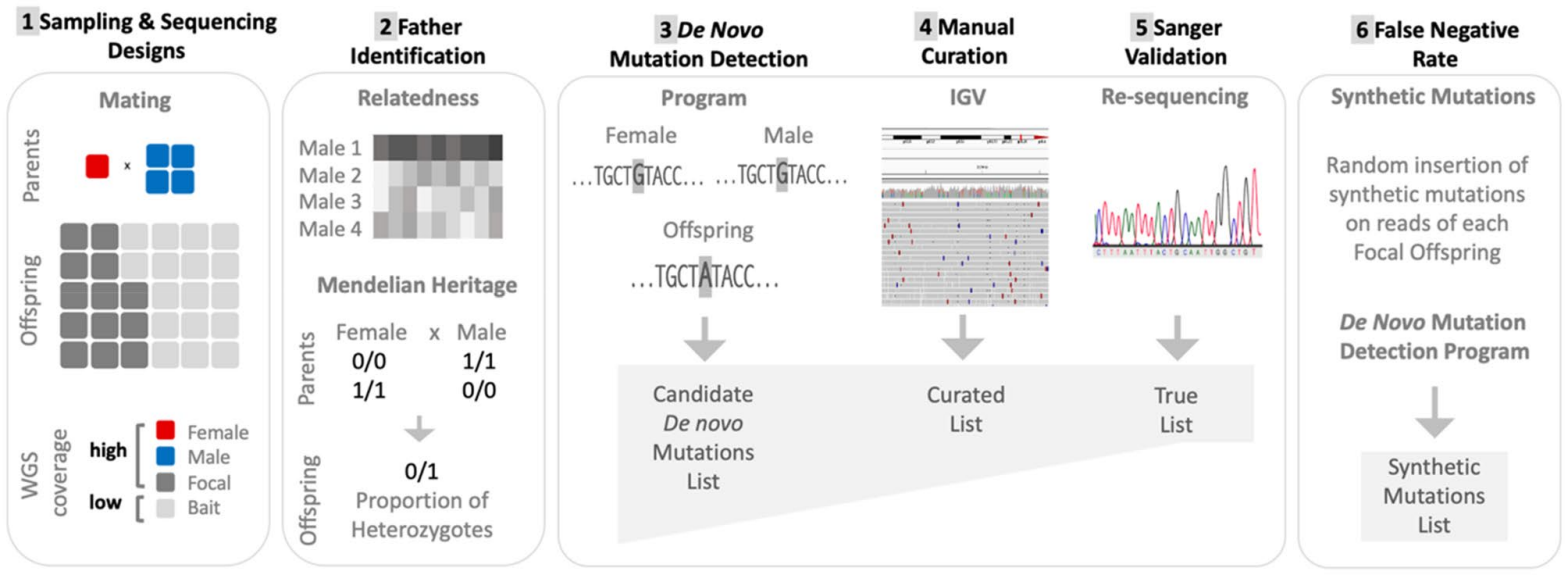
Experimental and analytical designs. Schematics of experimental and analytical procedures for direct mutation rate estimation which includes a series of parameters to exclude false positive mutations and account for false negatives. (1) Sampling & sequencing designs: experimental female and male mating, and whole genome sequencing of parents and offspring at high and low coverage levels. (2) Father identification: two methods are represented (relatedness and Mendelian heritage). (3) De novo mutation detection: candidate de novo mutation is absent from both parents. (4) Manual curation: Integrated Genome Viewer (IGV) for mapping visualization of reads of candidate mutation list. (5) Sanger validation: PCR amplification and re-sequencing of candidate mutation. (6) False Negative Rate (FNR): 1000 synthetic mutations were inserted in each focal offspring; reads are processed and the de novo mutation detection program performed as in (3).

The workflow for this study, depicted schematically in Fig. 3, consisted of sample collection, high-throughput genome sequencing, variant calling, confirmation of paternity, detection of putative de novo mutations in offspring, verification of mutations, and estimation of the false negative rate by use of simulated mutations.

### Mosquitoes

*Anopheles coluzzii* (MOPTI strain; MRA-763) and *A. stephensi* (STE2 strain; MRA-128) mosquito eggs were obtained from the Malaria Research and Reference Reagent Resource Center (BEI Resources, VA, USA) and reared at 26 °C and 80% relative humidity for multiple generations prior to experimental setup. Pupae were separated by sex and distributed in replicates with varying ratios of multiple males to single females to achieve mating. Adults were allowed to mate for four days before blood-feeding on heparin-treated bovine blood using a Hemotek artificial blood feeder (Hemotek Ltd, UK). Egg cups were placed in each mating cage after blood-feeding and hatched larvae were counted and transferred to a larger container for subsequent rearing. Replicates chosen for further analysis (one for each species) were those with the fewest F0 males but producing more than 30 F1 individuals, which were reared to adulthood, sorted by sex, and stored in 80% ethanol at −20 °C until DNA extraction.

### Whole genome sequencing

For each species, the genomes of the female, potential male parents, and a subset of the offspring (designated ‘focal’) were sequenced at a target depth of 30×, while the remaining offspring (designated ‘bait’) were sequenced at a lower target depth of 15×. This reflects a strategy to minimize false positive mutations by dividing the offspring into two arbitrary classes: focal and bait. Only sites in focal offspring were checked for candidate mutations, and the sites checked included only those without variants in the bait offspring. This resulted in an efficient filtering method to detect sites in the genome that are prone to erroneous read alignment. Since mis-mapping errors are expected to be frequent at the sites where they occur, they should be detected even at the lower depth of coverage employed for bait samples, thus economizing on sequencing resources.

Prior to DNA extraction, the lower abdominal segment of females (containing the spermathecae) were removed to prevent sperm DNA contamination in downstream sequencing analysis. DNA extraction was carried out using a Qiagen Biosprint 96 extraction robot (Qiagen, Hilden, Germany) following a protocol established in our lab for increased DNA yield^55^. Samples were quantified using a Qubit 2.0 Fluorometer (Life Technologies, CA, USA) and prepared for Illumina sequencing using 10 ng DNA input following the KAPA HyperPlus Prep kit manufacturer’s protocol (KAPA Biosystems, MA, USA) with minor modifications^56^. Resulting DNA libraries were pooled and submitted for sequencing as 150 bp paired-end reads on the Illumina HiSeq 4000 platform at the UC Davis Genome Center.

### Pre-processing of sequence data, mapping, variant calling

Low-quality and adapter sequences were removed from reads using Trimmomatic v0.39^57^ with settings illuminaclip:2:30:10, leading:3, trailing:3, slidingwindow:4:15, minlen:36. Reads were marked for PCR duplicates using Sambamba v0.6.7^58^. The trimmed paired reads of both species were mapped onto their respective reference genomes using BWA MEM v0.7.17^59^ with default settings. *Anopheles gambiae* reference genome ‘PEST AgamP4^60,61^ was the mapping target for *A. coluzzii* samples. The PEST genome originates from an *A. gambiae*-*A. coluzzii* hybrid laboratory strain and is commonly regarded as suitable for genomic analysis of both species^62,63^. The sequence reads of *A. stephensi* were mapped onto the recently assembled genome of *A. stephensi* ‘Indian’ strain^64^. Mapping quality control statistics were generated with Qualimap v2.2.1^65^. Joint variant calling was performed using Freebayes v1.1.1^66^ and biallelic SNPs were extracted from the VCF file using BCFtools v1.9^67^.

### Male parent identification

Various inheritance-based approaches are available for kinship analysis based on identification of common alleles shared by descent^68–70^. Here, we adopted similar approaches, using autosomal biallelic variant sites to identify the male parent from among potential fathers in each grouping by applying two strategies: (i) relatedness levels between parents and offspring were estimated by the KING inference method^43^ as implemented in –relatedness2 from VCFtools^71^; and (ii) concordance with expectations of Mendelian inheritance were assessed at variant sites where the genotypes of the parents are homozygous for different alleles, and therefore the offspring are expected to present only heterozygous genotypes. For the second method, separate analyses were performed for each potential male parent in combination with the female parent using a custom Perl script. The pair with the highest number of sites that were heterozygous in all offspring was identified as the true mating pair. After identification of the true fathers, the irrelevant F0 male samples were removed from the dataset.

### Calling candidate mutations

Starting with the biallelic SNPs for each species in VCF format we applied a defined program in Perl to filter each variant site according to a set of criteria which define a candidate mutation as follows:

- (i) Both parents are homozygous for the reference allele with read depth (DP) ≥ 10, and no alternate allele reads present (AD_ALT_ = 0).
- (ii) All bait offspring with called genotypes are homozygous for the reference allele with no alternate allele reads present (AD_ALT_ = 0).
- (iii) All focal offspring have a called genotype and either 1 or 2 focal offspring are called as heterozygotes (or are hemizygous for the alternate allele in the case of male offspring at sites on the X chromosome) and DP ≥ 10, while the remaining focal offspring are homozygous for the reference allele with no alternate allele reads present (AD_ALT_ = 0).
- (iv) For the heterozygous focal offspring in (iii): at sites on the autosomes, and for female offspring at sites on the X chromosome the number of alternate allele reads present (AD_ALT_) must be related to the total read depth (DP): 0.27 × DP ≤ AD_ALT_ ≤ 0.73 DP.

### Manual curation of candidate mutations

All candidate mutations detected by our program were examined using Integrated Genome Viewer (IGV)^72^ which facilitates visualization of aligned reads from the original BAM files. In order to rule out obvious mis-mapping artifacts candidate mutations were considered within the context of all overlapping reads and adjacent reads and their variant sites.

### Sanger sequencing

After manual curation with IGV, we evaluated all remaining candidates by Sanger sequencing to identify true mutations. For each candidate site, two or more sets of primers were designed to amplify the locus of interest from the DNA of parent–offspring trios (the mutated individual and both parents). Primers were designed based on the reference genome sequence of each species using Primer-BLAST^73^. Polymerase Chain Reaction (PCR) performed in an ABI Veriti thermal cycler (Applied Biosystems, USA) in 25 µl reaction volume containing: 12.5 µl 2 × GoTaq Green Master Mix (Promega), 1 µl of 10 pmol/µl of each forward and reverse primer, 9 µl of nuclease free water, and 1.5 µl of template DNA. Thermocycle conditions for the reaction were as follows: initial denaturation of 95 °C for 5 min, followed by 35 cycles of denaturation at 95 °C for 30 s, annealing at 55 °C for 30 s, with 30 s of extension, and a final extension of 72 °C for 5 min. Amplicons were cleaned using 1.8 × SPRI magnetic beads and sequenced on both strands at the UCDNA Sequencing Facility (Davis, CA, USA) using BigDye Terminator v. 3.1 chemistry on an ABI Prism 3730 Genetic Analyzer (Applied Biosystems, USA). Chromatograms were analyzed for each member of a trio to verify the presence of the mutation in the offspring but not in either parent. Genomic annotations for each mutation site were obtained from the published general feature format (GFF) files of *A. coluzzii*^74^ and *A. stephensi*^64^.

### Callable sites

The great majority of genomic sites are invariant within the pedigrees sequenced, and thus are not captured in the output of joint variant calling. On the other hand, not all sites absent from the variant calling results can confidently be assumed to be invariant. At sites with very low or no read coverage, a mutation will not be detected even when present. We reasoned that a read coverage of 10× will allow variant detection. Thus, to obtain an estimate of callable sites we defined the sets of all genomic coordinates with aligned read depth greater than 10× in each parent and each focal offspring, then intersected these three sets for each parent–offspring trio. A minority of sites reported by the joint variant caller were removed from the result for each trio since these sites (all except the handful of final candidate mutations) by failing our filtering regime had all proven to be un-callable by our methods. The remaining callable sites for each trio were summed to give the total number of callable genomic coordinates across all focal offspring. The final number of callable sites was doubled to account for the presence of diploid chromosomes.

### Synthetic mutations

To estimate the false negative rate (type 2 error), we added 1000 faux (= synthetic) mutations to each focal offspring sequence data and measured the ability of our analytical pipeline to detect them^19^. Synthetic mutations were randomly inserted in autosomal sites using BAMSurgeon software^75^. The introduction of a mutation was performed by changing a fraction of reads overlapping the site and replacing the existing base with an alternate base randomly selected for the site. The number of reads altered for each synthetic mutation was determined based on an empirical distribution of alternate allele read depths at all heterozygous autosomal sites in the full dataset for all offspring for each species. These empirical distributions were calculated for each overall read depth between 10 and 100. When a synthetic mutation was created at a site with a given overall read depth, the quantity of alleles altered was drawn from the appropriate, corresponding distribution for that depth. To calculate empirical distributions, determination of heterozygosity relied on the principles of Mendelian inheritance: when each parent was homozygous for a different allele, all offspring were assumed to be heterozygous at that site. For example, for *A. coluzzii* there were 82,616 such heterozygous sites where overall read depth was 12 (Supplementary Table 5). The empirical distributions for alternate allele depth at these sites, as well as the corresponding binomial distribution calculated from the data mean and variance, are shown in Supplementary Fig. 3. For a synthetic mutation site with depth 12, a random integer N would be chosen from the empirical frequency distribution and N reads would be changed to the randomly chosen alternate base in the focal individual’s BAM file. After introduction of synthetic mutations, all BAM files were converted into FASTQ format and then processed with the mapping, variant calling, and filtering methods described above. For each species, dividing the number of synthetic mutations detected by the number of synthetic mutations inserted into callable sites provided an estimate of the false negative rate (FNR) which was used as a correction factor in the final mutation rate calculation.

### Estimation of mutation rate

For the calculation of the mutation rate (µ), we desire the number of mutations per genome per generation. The enumerated total of mutations we detected in each species serves as the numerator. For the denominator we require an estimate of the number of callable sites, that is sites where we can confidently determine the presence or absence of a mutation, should one be present. For each species the corrected mutation rate was calculated as follow:

$$\mu =\frac{mutations \; detected}{callable \; sites}\times \frac{synthetic \; mutations \; inserted}{synthetic \; mutations \; detected}$$

## Supplementary Information

Supplementary Information.

## Data Availability

Whole genome sequence data included in this study are deposited in NCBI GenBank under BioProject PRJNA732889. *Anopheles stephensi* from accession number SAMN19349627–SAMN19349659 and *A. coluzzii* SAMN19355128–SAMN19355162.

## Code availability

Custom codes used for the analysis are available on GitHub page: https://github.com/vectorgenetics-lab/mutation-rate.
