# Supplementary material for: Spontaneous mutation rate estimates for the principal malaria vectors Anopheles coluzzii and Anopheles stephensi
Source: Sci Rep. 2022 Jan 7;12:226. doi: 10.1038/s41598-021-03943-z (PMC8742016; doi:10.1038/s41598-021-03943-z)
Supplement: Supplementary file 1 — Supplementary Information. [file 41598_2021_3943_MOESM1_ESM.pdf]

Supplementary Material

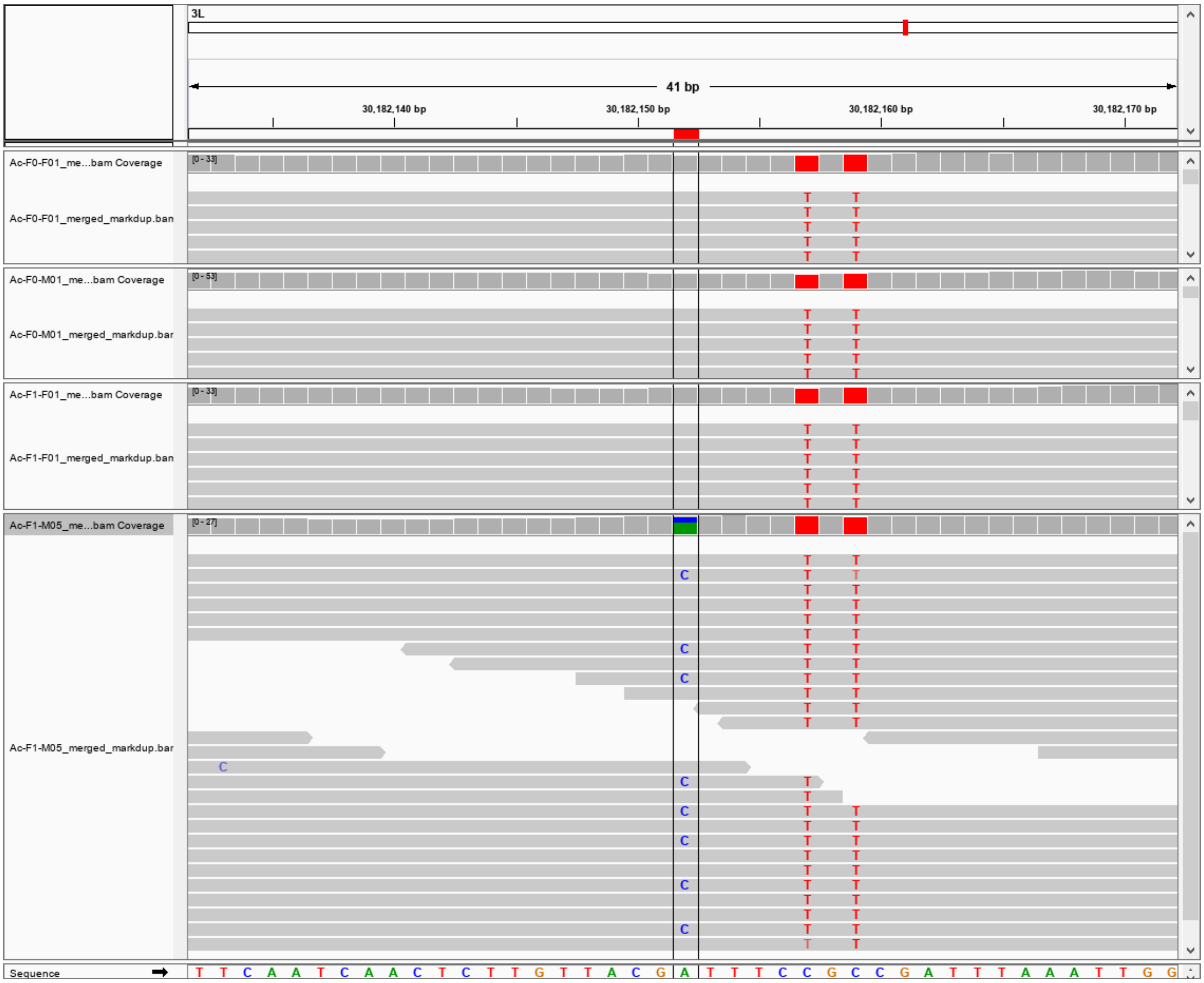

Supplementary Fig. 1 IGV screenshot of acceptable *de novo* mutation candidate example. Aligned sequence reads are visible as grey horizonal bands. Variant nucleotides are depicted in color at their respective positions. The top two panels show aligned reads of parents. A pair of central vertical lines encloses the identified mutation in a focal offspring (Ac-F1-M05), on chromosome 3L. The mutation is unique to the focal offspring in question and does not segregate with any adjacent substitution/indel on the same reads. Outside the center lines are two C→T substitutions homozygous in both parents and offspring.

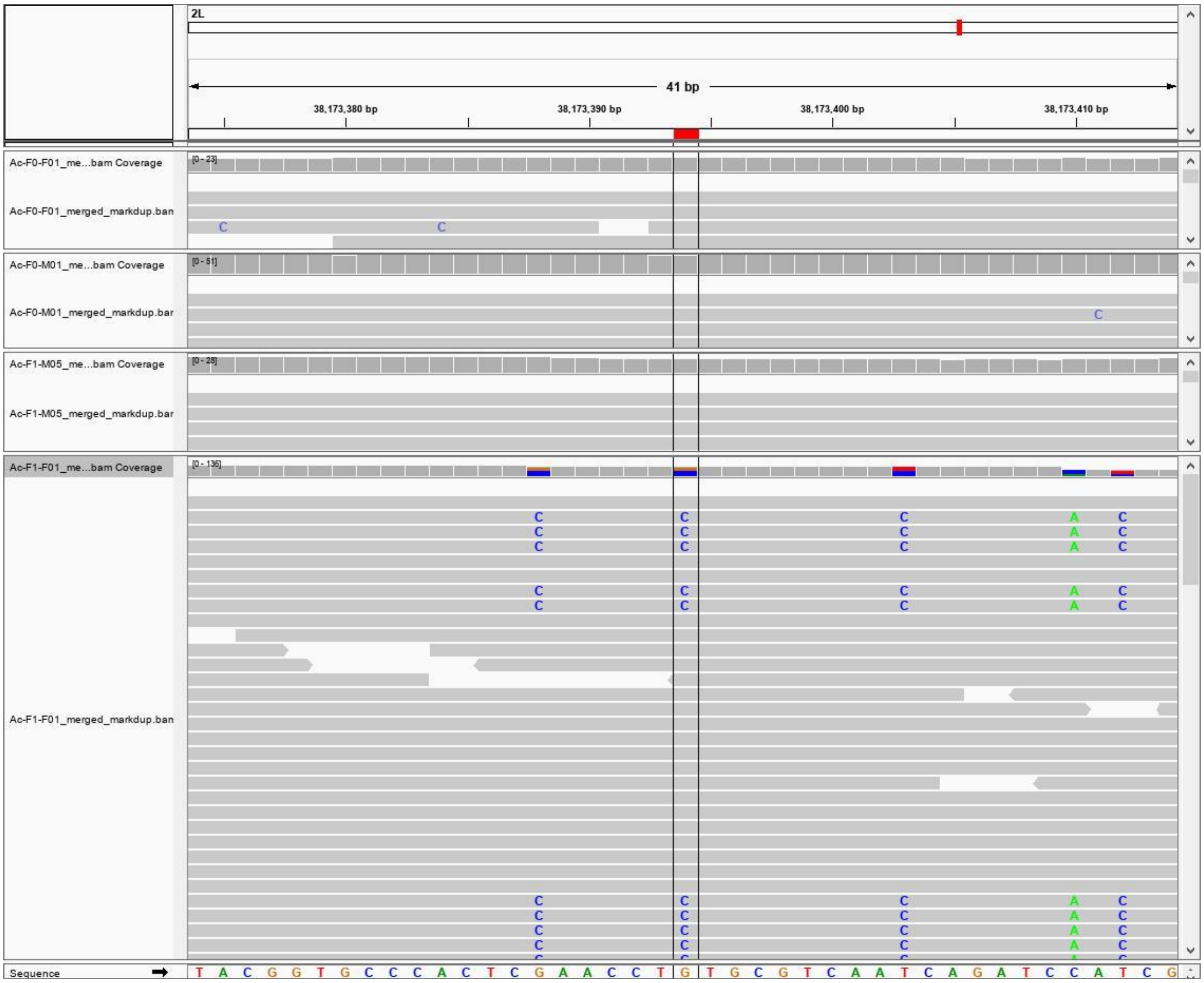

Supplementary Fig. 2 IGV screenshot of unacceptable *de novo* mutation candidate example. Aligned sequence reads are visible as grey horizonal bands. Variant nucleotides are depicted in color at their respective positions. The top two panels show aligned reads of parents. A pair of central vertical lines encloses an identified mutation in a focal offspring (Ac-F1-F01) on chromosome 2L. There are multiple variant for this offspring on that genomic region that are not present on the parents, thus considered as artifacts and false positive.

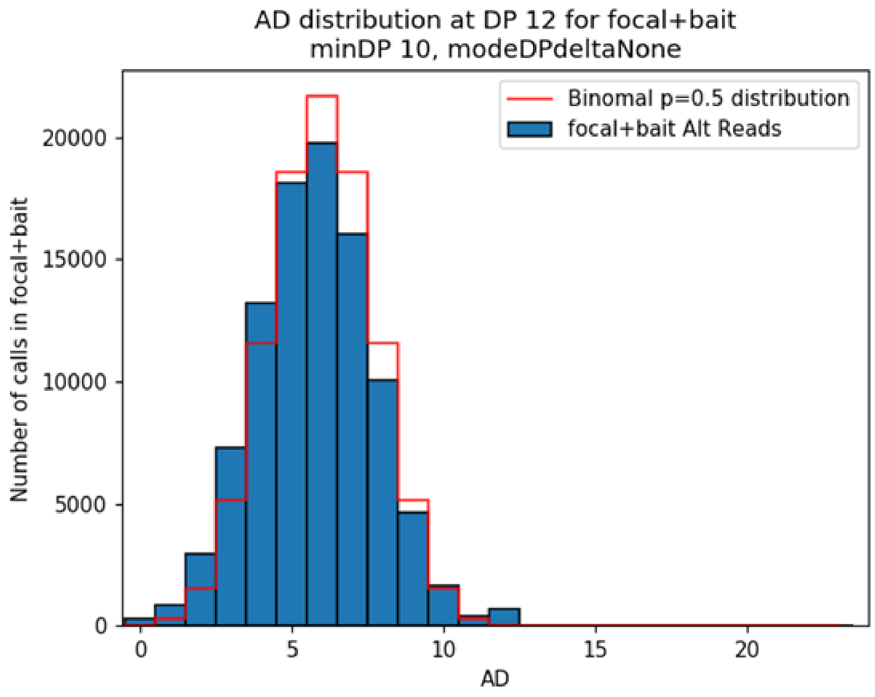

Supplementary Fig. 3 Alternate allele depth and binomial distributions. Example of distribution of number of sites in all offspring (focal + bait) showing different values of alternate allele depth (AD) for read depth (DP) = 12.

Supplementary Table 1. ***Anopheles coluzzii*** **whole genome sequencing information.**

Number of raw reads, mapped reads and NCBI accession number for each sample.

Sample Name	Raw Reads	Mapped Reads	% Mapped	Mean Coverage	Sample Type	Accession Number
Ac-F0-F01	97140913	45489817	46.83%	20.5963	parent	SAMN19355128
Ac-F0-M01	103341843	101637716	98.35%	45.1053	parent	SAMN19355129
Ac-F0-M02	60835790	60265105	99.06%	27.7418	parent	SAMN19355130
Ac-F0-M03	61386017	60521358	98.59%	27.2453	parent	SAMN19355131
Ac-F0-M04	69292077	67321258	97.16%	30.26	parent	SAMN19355132
Ac-F1-M12	52232611	51793031	99.16%	23.5697	focal	SAMN19355141
Ac-F1-F01	58181099	49312578	84.76%	22.6875	focal	SAMN19355153
Ac-F1-F08	73278387	72595327	99.07%	33.1159	focal	SAMN19355150
Ac-F1-F09	94273164	56766077	60.21%	25.602	focal	SAMN19355151
Ac-F1-F10	71110716	55405601	77.91%	25.1209	focal	SAMN19355152
Ac-F1-F13	110058282	1840775	1.67%	0.4933	focal	SAMN19355156
Ac-F1-M01	76915421	59766572	77.70%	27.1498	focal	SAMN19355157
Ac-F1-M02	67304960	66750852	99.18%	30.2942	focal	SAMN19355158
Ac-F1-M03	59789278	59305240	99.19%	27.1593	focal	SAMN19355159
Ac-F1-M04	51570146	51140260	99.17%	23.5429	focal	SAMN19355160
Ac-F1-M05	48474172	48056314	99.14%	22.0738	focal	SAMN19355161
Ac-F1-F14	41437727	28051181	67.69%	12.7125	bait	SAMN19355133
Ac-F1-F02	31522936	31079182	98.59%	14.2498	bait	SAMN19355145
Ac-F1-F11	87944465	20890255	23.75%	9.5483	bait	SAMN19355154
Ac-F1-F12	91105174	39712994	43.59%	17.8925	bait	SAMN19355155
Ac-F1-F15	35372035	31374599	88.70%	14.3567	bait	SAMN19355134
Ac-F1-F16	43488216	38829424	89.29%	17.6019	bait	SAMN19355135
Ac-F1-F17	22030445	21824248	99.06%	10.1544	bait	SAMN19355136
Ac-F1-F18	25738247	25466756	98.95%	11.8689	bait	SAMN19355137
Ac-F1-F19	39722485	39319605	98.99%	18.0755	bait	SAMN19355138
Ac-F1-F20	38087731	35954639	94.40%	16.4626	bait	SAMN19355139
Ac-F1-M06	27698935	27432414	99.04%	12.5688	bait	SAMN19355146
Ac-F1-M07	20557860	19095236	92.89%	8.881	bait	SAMN19355147
Ac-F1-M08	27903134	27445157	98.36%	12.761	bait	SAMN19355148
Ac-F1-M09	23811781	23565913	98.97%	10.9727	bait	SAMN19355149
Ac-F1-M10	65352121	21929765	33.56%	9.7912	bait	SAMN19355162
Ac-F1-M11	27270106	26897263	98.63%	12.3656	bait	SAMN19355140
Ac-F1-M13	37059248	36766383	99.21%	17.0231	bait	SAMN19355142
Ac-F1-M14	26938951	26712829	99.16%	12.3178	bait	SAMN19355143
Ac-F1-M15	35379886	35068829	99.12%	16.0302	bait	SAMN19355144

Supplementary Table 2. ***Anopheles stephensi*** **whole genome sequencing information.**

Number of raw reads, mapped reads and NCBI accession number for each sample.

Sample Name	Raw Reads	Mapped Reads	% Mapped	Mean Coverage	Sample type	Accession Number
As19-STE2-PF1	77776380	76593984	98.48%	40.7268	parent	SAMN19349657
As19-STE2-PM2	75436497	74687505	99.01%	38.5068	parent	SAMN19349659
As19-STE2-PM1	52404190	51855823	98.95%	27.9091	parent	SAMN19349658
As19-STE2-F01	66954375	66137779	98.78%	34.7842	focal	SAMN19349627
As19-STE2-F07	69430894	68597695	98.80%	35.8084	focal	SAMN19349629
As19-STE2-F12	70532115	69703538	98.83%	36.2458	focal	SAMN19349632
As19-STE2-F14	65718226	64881492	98.73%	34.3319	focal	SAMN19349633
As19-STE2-F18	57651886	57022285	98.91%	30.1536	focal	SAMN19349635
As19-STE2-F19	61559874	60847229	98.84%	32.1884	focal	SAMN19349636
As19-STE2-F23	67998247	67218696	98.85%	35.3671	focal	SAMN19349640
As19-STE2-M02	78965451	78427509	99.32%	41.2406	focal	SAMN19349644
As19-STE2-M08	88316059	87728363	99.33%	46.034	focal	SAMN19349648
As19-STE2-M09	64077766	63573092	99.21%	33.169	focal	SAMN19349649
As19-STE2-M12	77656885	77106199	99.29%	41.0428	focal	SAMN19349652
As19-STE2-M15	73758339	73257163	99.32%	39.0162	focal	SAMN19349655
As19-STE2-M16	67535981	67032143	99.25%	35.2754	focal	SAMN19349656
As19-STE2-F06	31303280	30901421	98.72%	15.8275	bait	SAMN19349628
As19-STE2-F08	31135114	30751733	98.77%	15.7036	bait	SAMN19349630
As19-STE2-F11	31404047	30941221	98.53%	15.978	bait	SAMN19349631
As19-STE2-F15	31392254	30968507	98.65%	16.0192	bait	SAMN19349634
As19-STE2-F20	29725268	29348859	98.73%	15.0866	bait	SAMN19349637
As19-STE2-F21	29588753	29216355	98.74%	15.3141	bait	SAMN19349638
As19-STE2-F22	30199414	29830130	98.78%	15.4727	bait	SAMN19349639
As19-STE2-F24	30484601	30110987	98.77%	15.6666	bait	SAMN19349641
As19-STE2-F25	30048552	29673296	98.75%	15.2886	bait	SAMN19349642
As19-STE2-M01	34959253	34664794	99.16%	18.0344	bait	SAMN19349643
As19-STE2-M04	30760393	30517098	99.21%	15.8295	bait	SAMN19349645
As19-STE2-M05	38277837	37784715	98.71%	20.1156	bait	SAMN19349646
As19-STE2-M07	31517930	31317055	99.36%	16.4468	bait	SAMN19349647
As19-STE2-M10	33896520	33633579	99.22%	17.6547	bait	SAMN19349650
As19-STE2-M11	32227024	32004439	99.31%	16.9569	bait	SAMN19349651
As19-STE2-M13	32302724	32006474	99.08%	16.9231	bait	SAMN19349653
As19-STE2-M14	35161264	34916379	99.30%	18.3509	bait	SAMN19349654

Supplementary Table 3. List of candidate *de novo* mutations detected in *A. coluzzii.* Genomic position, reference (Ref.) and alternate (Alt.) alleles, focal offspring where candidate mutation was detected, alternate allele frequency, allele and read depth mean for parents and focal offspring.

Chromosome, Position	Allele		Focal offspring	Alt. Frequency	Allele Depth		Read Depth Mean	
	Ref.	Alt.			Ref.	Alt.	Parents	Focal
2L, 1679821	C	T	F1-F01	0.308	9	4	55.0	31.4
2L, 21501855	A	G	F1-M01	0.273	16	6	35.0	28.1
2L, 38173388	G	C	F1-F01	0.527	26	29	30.0	31.9
2L, 38173394	G	C	F1-F01	0.527	26	29	30.0	31.6
2L, 38173403	T	C	F1-F01	0.491	28	27	30.5	30.3
3L, 10978191	G	A	F1-M04	0.529	8	9	31.5	26.9
3L, 30182152	A	C	F1-M05	0.348	15	8	31.5	28.2
3L, 32059779	C	T	F1-F09	0.389	11	7	26.5	17.8
**All site's Mean**				**0.424**			**33.8**	**28.3**

Supplementary Table 4. List of candidate *de novo* mutations detected in *A. stephensi.* Genomic position, reference (Ref.) and alternate (Alt.) alleles, focal offspring where candidate mutation was detected, alternate allele frequency, allele and read depth mean for parents and focal offspring.

Chromosome, Position	Allele		Focal Offspring	Alt. Frequency	Allele Depth		Read Depth Mean	
	Ref.	Alt.			Ref.	Alt.	Parents	Focal
X, 1692026	C	T	As19-STE2-F23	0.3077	18	8	22.0	18.62
X, 14920753	C	T	As19-STE2-M15	1	0	12	27.0	17.85
2, 29186821	T	A	As19-STE2-M09	0.5588	15	19	39.5	30.85
2, 42294046	C	A	As19-STE2-M12	0.2973	26	11	30.0	27.38
3, 6181251	G	C	As19-STE2-M02	0.5185	13	14	26.5	26.31
3, 56020205	G	T	As19-STE2-F14	0.4737	10	9	35.5	27.15
**All site's mean**				**0.562**			**30.1**	**24.7**

Supplementary Table 5. Site distribution by read depth in *A. coluzzii*. Empirical distribution of number of sites by read depth ranging from 2 to 100.

Depth	Sites	Depth	Sites
2	1171	48	701
3	4384	49	537
4	10254	50	386
5	19351	51	309
6	30821	52	243
7	43500	53	174
8	56178	54	130
9	66656	55	74
10	74891	56	64
11	79701	57	59
12	82616	58	37
13	82162	59	31
14	79924	60	20
15	76352	61	21
16	71500	62	8
17	66657	63	10
18	62090	64	2
19	57638	65	3
20	53817	66	6
21	50530	67	4
22	46591	68	6
23	43938	69	3
24	41322	70	4
25	38727	71	3

26	36204	73	4
27	33337	74	3
28	30440	76	1
29	28159	77	1
30	25392	79	1
31	22701	80	1
32	19948	82	1
33	17734	83	1
34	15308	84	4
35	13017	86	2
36	11092	87	2
37	9279	88	2
38	7675	89	2
39	6483	90	3
40	5202	91	5
41	4290	93	3
42	3502	94	1
43	2708	95	3
44	2138	96	4
45	1590	98	1
46	1236	99	5
47	925	100	4

Supplementary Table 6. Summary of synthetic mutation analysis and estimation of mutation rates for *A. coluzzii* and *A. stephensi*. Synthetic mutations (SMs).

**Category**		***Anopheles coluzzii***	***Anopheles stephensi***
SMs introduced		10000	13000
SMs in biallelic sites		8903	11525
SMs in reference homozygous parents (i)		8788	11453
SMs in pure sites and called in all focal offspring (ii, iii)		8070	10014
SMs detected		7662	9563
Haploid callable sites		1945418861	2439887457
Estimated mutation rate (μ)		1.01×10^{-9}	1.39×10^{-9}
95% confidence level	Lower	2.07×10^{-10}	4.51×10^{-10}
	Upper	2.93×10^{-9}	3.25×10^{-9}
